# Negative emotion modulates prefrontal cortex activity during a working memory task: a NIRS study

**DOI:** 10.3389/fnhum.2014.00046

**Published:** 2014-02-10

**Authors:** Sachiyo Ozawa, Goh Matsuda, Kazuo Hiraki

**Affiliations:** Graduate School of Arts and Science, The University of Tokyo, JST, CRESTTokyo, Japan

**Keywords:** NIRS, prefrontal activity, DLPFC, cognitive control, emotion regulation, working memory, n-back task, IAPS

## Abstract

This study investigated the neural processing underlying the cognitive control of emotions induced by the presentation of task-irrelevant emotional pictures before a working memory task. Previous studies have suggested that the cognitive control of emotion involves the prefrontal regions. Therefore, we measured the hemodynamic responses that occurred in the prefrontal region with a 16-channel near-infrared spectroscopy (NIRS) system. In our experiment, participants observed two negative or two neutral pictures in succession immediately before a 1-back or 3-back task. Pictures were selected from the International Affective Picture System (IAPS). We measured the changes in the concentration of oxygenated hemoglobin (oxyHb) during picture presentation and during the n-back task. The emotional valence of the picture affected the oxyHb changes in anterior parts of the medial prefrontal cortex (MPFC) (located in the left and right superior frontal gyrus) and left inferior frontal gyrus during the n-back task; the oxyHb changes during the task were significantly greater following negative rather than neutral stimulation. As indicated in a number of previous studies, and the time courses of the oxyHb changes in our study, activation in these locations is possibly led by cognitive control of emotion, though we cannot deny it may simply be emotional responses. There were no effects of emotion on oxyHb changes during picture presentation or on n-back task performance. Although further studies are necessary to confirm this interpretation, our findings suggest that NIRS can be used to investigate neural processing during emotional control.

## Introduction

A number of previous studies have suggested that emotion is regulated by cognitive processing (Dolcos et al., 2011; Cromheeke and Mueller, 2013). Studies of the cognitive control of emotion or emotion regulation have utilized neuroimaging methods, which are a useful tool that provide objective and precise indices of subjective emotional experiences. A large number of neuroimaging studies have recently been conducted in order to examine the neural functions underlying the cognitive control of emotion (Hare et al., 2005; Erk et al., 2006; Goldin et al., 2008; Adrian et al., 2011). According to these neuroimaging studies, the neural function of emotion regulation involve interactions between the *hot* emotional system in the limbic regions that involves the amygdala and *cold* higher order systems in prefrontal regions (Gray et al., 2002; Dolcos et al., 2011). More concretely, emotional control involves neural interactions that decrease the activity in the amygdala while increasing prefrontal activity. For example, Erk et al. (2006) have shown that working memory tasks downregulate amygdala activity induced by the anticipation of subsequent negative emotions. Ochsner et al. (2002) have indicated that amygdala activity is downregulated by reappraisals, when participants willfully no overrode their negative feelings toward the pictures. Van Dillen et al. (2009) have demonstrated that arithmetic tasks with a higher cognitive load downregulate the increased amygdala activity induced by a prepresented negative stimulus, and release negative emotions compared to arithmetic tasks with a lower cognitive load. Thus, cognitive processing associated with prefrontal enhancements has been shown to decrease activity in the limbic system. At the same time, the negative emotional state is thought to be relieved.

The cold, higher order, system includes brain regions typically associated with executive functions (Dolcos et al., 2011). One of these executive functions is working memory, which contributes to the maintenance of information relevant to current tasks over short periods of time (Baddeley and Della Sala, 1996). During the engagement of a working memory task, brain activity increases in frontal regions that include the dorsolateral prefrontal cortex (DLPFC) (Owen et al., 2005). Moreover, a number of studies have indicated that the cognitive control of emotion also activates various prefrontal regions, such as the orbitofrontal cortex (OFC; Banks et al., 2007), the medial prefrontal cortex (MPFC; Ochsner et al., 2002), and the ventrolateral prefrontal cortex (VLPFC; Lévesque et al., 2003). In particular, activity in the DLPFC has been consistently observed in a number of studies (Beauregard et al., 2001; Lévesque et al., 2003). DLPFC activity has been thought to be indirectly and reciprocally associated with activity in limbic systems through connections in the OFC (Cavada et al., 2000). The DLPFC appears to be the shared region for both working memory and emotional regulation.

In this study, we investigated whether task-irrelevant emotional stimuli elicited differential neural activity during a subsequent working memory task in order to investigate the prefrontal activity underlying the cognitive control of emotion. Previous findings (Dolcos et al., 2011) have suggested that task-irrelevant emotional stimuli can interfere with the maintenance of goal-relevant information and thus serve as a distracter in a cognitive task. A distracter can be presented during (Dolcos and McCarthy, 2006) or prior to (Deckersbach et al., 2008; Van Dillen et al., 2009; Hart et al., 2010) a working memory task. According to Dolcos and McCarthy (2006), when an emotional distracter is presented during a working memory task, DLPFC activity may be attenuated by the distracter and cognitive performance may be impaired. Only a few studies have been conducted with a prepresented distracter. For example, Van Dillen et al. (2009) have found greater activity in the right DLPFC in response to negative pictures than neutral pictures during an arithmetic task following 4 s of emotional picture presentation. A higher cognitive load also elicited greater activity in the right DLPFC. In addition, Hart et al. (2010) have examined the effects of emotional priming on a subsequent Stroop task. In that experiment, a negative or neutral picture was primed for 150 ms, and a Stroop task was then presented. Consequently, an attenuating effect was observed in response to the negative pictures during a congruent condition, but it was not observed during an incongruent condition. Hart et al. (2010) have explained that the need for cognitive processing overrode the deactivating effect of negative stimuli. As mentioned above, Van Dillen et al. (2009) have reported prefrontal activation during a cognitive task after emotional stimulation, and this was in contrast to Hart et al. (2010). The discrepancy of their results may partially depend on the duration of emotional stimulation. On the other hand, both of these studies have reported more activation in the prefrontal cortex during a task with a high cognitive load than that with low cognitive load. Therefore, emotional stimulation for at least 4 s, as in Van Dillen et al. (2009), and a task with a high cognitive load seem to be necessary to investigate the neural correlates of the cognitive control of emotion.

In order to measure prefrontal activity, we employed near-infrared spectroscopy (NIRS). The attachment of the NIRS system is easy, and it imposes little strain on the participants. If the cognitive control of emotion can be captured by NIRS, emotion regulation studies will be easily conducted in a wide variety of participants, including psychiatric patients and children, without imposing a strain on them. Although NIRS is unable to measure deep activity in the brain, such as in the limbic system, several NIRS studies have recently attempted to capture emotion-related activity in prefrontal regions (Herrmann et al., 2008; Dieler et al., 2010; Hoshi et al., 2011). For example, Hoshi et al. (2011) have observed that strong unpleasant pictures activate the bilateral VLPFC, while strong pleasant pictures deactivate the left DLPFC. Dieler et al. (2010) have shown more activation in the right DLPFC and right VLPFC during attempts to suppress thoughts with negative content than those with neutral or positive contents.

In our experiment, negative and neutral pictures were selected from the International Affective Picture System (IAPS; Lang et al., 1998). In order to induce strong emotional response, a series of two pictures of the same emotional valence were presented in succession for approximately 10 s. For cognitive processing, an n-back working memory task was employed. The n-back task is one of the most prevalent paradigms used in the assessment of working memory. We employed two levels of cognitive load (a high cognitive load or a low cognitive load) because cognitive load can modulate the processing of negative emotion (Erk et al., 2007; Van Dillen et al., 2009). Thus, in the experiment, participants observed two negative or two neutral pictures in succession and then performed a 1-back or 3-back task. Analyses of hemodynamic responses were conducted independently for the picture presentation and n-back task.

We anticipated that, during picture presentation, prefrontal activation would be significantly greater in response to negative pictures than in response to neutral pictures in line with the results of Hoshi et al. (2011). For the n-back task period, the prefrontal region that includes the DLPFC would be activated after negative stimulation relative to neutral stimulation. As mentioned above, the DLPFC and other prefrontal regions have been shown to exhibit general activation during the cognitive control of emotion (Beauregard et al., 2001; Lévesque et al., 2003). In addition, because a previous study has reported that a high cognitive load downregulates amygdala activity significantly more than a low cognitive load (Van Dillen et al., 2009), we anticipated that cognitive processing with a high cognitive load (3-back) would increase prefrontal activity significantly more than that with a low cognitive load (1-back). The cognitive performance may be impaired in response to the distraction of negative emotional stimulation (Dolcos and McCarthy, 2006).

## Materials and methods

### Participants

Twenty healthy male undergraduate students (mean age: 19.38 ± 0.79 years) participated in this study. All participants were right-handed. None of them had with any history of psychiatric or neurological disorders. The study conformed to the Declaration of Helsinki and was approved by the Ethics Committee of the University of Tokyo. All participants were given a comprehensive explanation of the experimental procedures by an experimenter, and they provided written informed consents for participation.

### Experimental environment

The experiment was performed in a shielded room. Participants were instructed to sit on a chair in front of a 17-in Cathode Ray Tube monitor (32 × 24 cm). The distance between a participant and the monitor was set to around 80 cm. The NIRS equipment (Spectratech OEG-16; Spectratech Inc., Yokohama, Japan) was attached to their heads. The experimental task was implemented in dim light.

### Emotional stimuli

Thirty-two neutral and 32 negative pictures were selected from the IAPS (Lang et al., 1998) based on a 9-point rating scale of the IAPS normative data. The average emotional valence rating of the selected pictures was 5.10 for the neutral pictures and 2.00 for the negative pictures, and the average arousal rating was 3.18 for the neutral pictures and 6.00 for the negative pictures. The arousal ratings of all of the selected negative pictures were above 5.5. Examples of the neutral pictures selected were humans, plants, food, and materials, and examples of the negative pictures were victims, mutilations, insects, and dirty toilets. Pictures with obscure content were not selected.

### n-back task

The 1-back and 3-back tasks were implemented with the digits 1–9. White-colored digits with a size of 12.70 mm were individually displayed in the middle of the black background, in sequence. The participants were instructed to press the Enter key as quickly as possible only when the current digit matched the one from one step or three steps earlier for the 1-back or 3-back, respectively. A total of 13 digits were presented in a sequence with 3 matches included.

### Experimental procedures

At the beginning of the experiment, participants were instructed to avoid head and body movements and deep breathing as much as possible during the NIRS measurement. The state of the participants was video-monitored and no salient head and body movement or deep breathing was detected.

**Figure 1:**
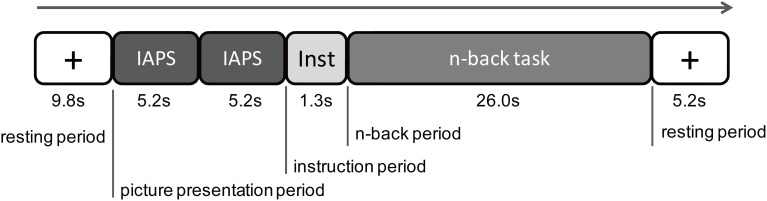
**Procedure of one experimental trial.** There were five periods in one trial: a first resting period, a picture presentation period, an instruction period, an n-back period, and then a resting period. During the picture presentation period, two International Affective Picture System (IAPS) pictures of the same emotional valence were presented. During the instructional period, a letter of the 1-back task or the 3-back task was displayed on the screen. During the n-back task period, the 1-back task or 3-back task was performed by the participant.

The experiment consisted of two successive sessions: a NIRS measurement session and a valence rating session. First, the NIRS equipment was attached to the participants' heads. Each trial in the NIRS measurement session included five periods (Figure 1). In the first resting period, a white fixed cross was displayed in the middle of the screen for 9.8 s. The participant was instructed to gaze at it calmly. In the picture presentation period, two IAPS pictures with the same emotional valence (neutral or negative) were sequentially presented for 5.2 s per picture without an interval. Soon after the picture presentation, white letters that read *1-back task* or *3-back task* were presented on the black background for 1.3 s (instruction period). In the n-back period, a sequence of digits was presented for 26.0 s. Each digit was presented on the screen for 300 ms, which was followed by a 1,700-ms interstimulus interval. After the n-back period, a fixed cross was presented for 5.2 s (the last resting period). There were six practice trials with only neutral pictures and 32 experimental trials with both neutral and negative pictures in the NIRS measurement session. At the end, the NIRS equipment was removed, and the participant rated the emotional valence of all of the pictures used in the experiment with a digital scale with nine grades from 1 (unpleasant) to 9 (pleasant).

### Behavioral analysis

The accuracy and reaction time of the n-back task were used as parameters in the behavioral analysis. The accuracy was determined by the percentage of correct responses. The reaction time was the time from the disappearance of a digit to the participants pressing the Enter key.

In order to examine the effects of emotion on the performance of the cognitive task, a 2 (emotional valence: neutral or negative) × 2 (cognitive load: 1-back or 3-back) repeated measures ANOVA with within-subjects factors was performed.

### Near-infrared spectroscopy recording and analysis

#### Near-infrared spectroscopy recording

**Figure 2:**
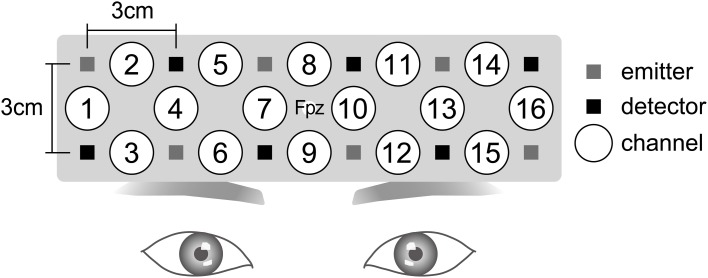
**Near-infrared spectroscopy (NIRS) settings.** The Spectratech OEG-16 NIRS system consisted of six emission probes and six detection probes. Sixteen channels existed. For the attachment of the NIRS system, the center of the probe holder was placed on Fpz in the International 10/20 System.

We used the Spectratech OEG-16, which consisted of six emission probes and six detection probes. This system is able to obtain three parameters of the concentration changes of hemoglobin: oxygenated (oxyHb), deoxygenated (deoxyHb), and total (totalHb) changes. The measures of the hemoglobin changes were obtained from the 16 channels. The emission probe and detection probe were 3 cm apart from each other, and all of the probes were set in a 15 × 3-cm matrix area. The Spectratech OEG-16 employs two wavelengths, which are approximately 770 and 880 nm, to record the absorption changes of hemoglobin at a depth approximately 2 cm below the scalp (Watanabe et al., 2000). The sampling interval was 655 ms. The center of the probe holder was placed on Fpz in the International 10/20 System (Figure 2). Our measurement area covers Fp1, Fpz, Fp2, F7, and F8, and regions a little inferior to F3 and F4. According to Okamoto et al. (2004), Fp1 and Fp2 are located on the left and right superior frontal gyrus, respectively. F7 and F8 are placed in the left and right inferior frontal gyrus, respectively. F3 and F4 are located in the inferior part of the DLPFC.

#### Near-infrared spectroscopy analysis

We examined the concentration changes of oxyHb in the analysis because it is the most sensitive measure to changes in regional cerebral blood flow (Hoshi et al., 2001), and it has the strongest correlation with blood-oxygen-level-dependent signals (Strangman et al., 2002).

The raw oxyHb data were high-pass filtered (0.0076 Hz). Each channel was analyzed individually. All sampling data was segmented to each trial and converted to a z-score with the mean value and the standard deviation (*SD*) of oxyHb change during the last 4.59 s (7 samples) of the first resting period used as a baseline. Thus, the mean and *SD* of the baseline were changed into the z-scores of 0 and 1 (Matsuda and Hiraki, 2006). For artifact rejection, all difference values between the sampling data and their *SD*s were calculated. The trials that included at least one difference value over ±8 *SD* were excluded from the statistical analysis.

The prefrontal activities during the picture presentation and n-back task were separately analyzed by conducting both individual and group analyses. In the picture presentations, the oxyHb changes resulting from the neutral or negative picture presentation were analyzed. For the n-back task, the oxyHb changes of the 1-back and 3-back tasks after neutral picture presentation (neutral 1-back, neutral 3-back, respectively) and 1-back task and 3-back task after negative picture presentation (negative 1-back, negative 3-back, respectively) were analyzed independently.

In individual analysis, the means and *SD*s of the oxyHb change in all trials were calculated in each of the picture presentations and the n-back task. Then, the mean oxyHb values were compared with the baseline, in which the average value was 0, with a one-sample *t*-test in order to determine any significant activation.

In the group analysis of picture presentation, the means and the *SD*s of the oxyHb changes of all participants were calculated. The mean oxyHb values were compared with baseline, in which the average value was 0, with a one-sample *t*-test. In addition, Student's *t*-test was performed between the mean oxyHb values of negative and neutral picture presentations to examine the differences in prefrontal activity between emotional valences.

In the group analysis of the n-back task, the means and *SD*s of oxyHb change of all participants were calculated. A 2 (emotional valence: neutral or negative) × 2 (cognitive load: 1-back or 3-back) repeated measures ANOVA with within-subjects factors was performed.

## Results

### Behavioral results

#### Rating of the emotional valence

In order to confirm whether the pictures used for the emotional stimulation in this study had significantly different emotional valence ratings, *t*-tests were performed. Neutral pictures were significantly different from negative pictures in their emotional valence ratings [mean valence scores: neutral, 4.21; negative, 1.96; *t*_(19)_ = 10.07, *P* < 0.001].

#### Behavioral performance

A 2 (emotional valence: neutral or negative) × 2 (cognitive load: 1-back or 3-back) repeated measures ANOVA revealed no main or interaction effect for accuracy (Table 1) and a significant main effect of cognitive load for reaction times. A *post-hoc* analysis showed that reaction times in the 1-back task were significantly faster than those in the 3-back task (mean reaction time: 1-back, 246.32 ms; 3-back, 363.77 ms, *P* < 0.001). There was no other main or interaction effect for reaction times.

**Table 1:** **Means and standard deviations of accuracy and reaction time of response**.

	**Neutral picture presentation**		**Negative picture presentation**	
	**1-back**	**3-back**	**1-back**	**3-back**
Accuracy (% correct)	94.58 (4.30)	90.83 (13.36)	94.79 (6.61)	88.75 (14.88)
Reaction time (ms)	241.46 (94.71)	354.83 (179.08)	251.18 (122.49)	372.71 (166.29)

### Near-infrared spectroscopy

#### oxyHb changes during picture presentation

The result of the individual analysis is shown in Table 2. For neutral picture presentations, significant oxyHb increases were observed in seven of 20 participants (35%) while significant oxyHb decreases was found in three participants (15%). For the negative picture presentations, there were nine of 20 participants (45%) who showed significant oxyHb increases while six participants (30%) showed significant oxyHb decreases. Channels that showed significant oxyHb changes were also inconsistent between participants.

**Table 2:** **Channels that showed significant oxyHb changes during picture presentation (individual analysis)**.

**Participant no.**	**Neutral picture presentation**		**Negative picture presentation**	
	**Activation**	**Deactivation**	**Activation**	**Deactivation**
1	-	-	-	3/4/6/12
2	-	-	2/9/12/16	-
3	-	-	-	-
4	2/8–11/14–16	-	-	-
5	-	-	-	-
6	-	16	-	16
7	-	1/3–6/9–11/13/15	8	-
8	1–16	-	1–16	-
9	-	-	-	-
10	-	-	-	-
11	-	-	7/11–15	5
12	-	-	-	-
13	-	-	1/3/14	-
14	2/4/6/8–15	-	2/9/12	16
15	2/5/9/11/14	-	1–11/13–16	-
16	1–9/11–15	-	2/3/8–11/14	-
17	-	3/15	-	2–5/11/14/15
18	12/15	-	-	3/16
19	-	-	-	-
20	8	-	15	-

Numerical numbers indicate channel numbers. The mark “-” indicates no significant oxyHb changes observed in any of the channels.

Table 3 displays the results of the group analysis. It represents the means and *SD*s of oxyHb changes of all participants in each channel. A one-sample *t*-test revealed significant activation in three channels during the negative picture presentation compared to baseline. In contrast, significant activation was found in 12 channels in response to the neutral picture presentation.

**Table 3:** **The oxyHb changes during picture presentation (group analysis)**.

**Channel no.**	**Neutral picture presentation**				**Negative picture presentation**			
	***M***	***SD***	***t*_(19)_**	***P***	***M***	***SD***	***t*_(19)_**	***P***
1	−0.01	0.90	−0.05	0.958	0.50	1.55	1.44	0.165
2	0.83	1.35	2.74	0.013^*^	0.94	2.29	1.85	0.080
3	0.56	1.66	1.51	0.148	0.57	2.69	0.95	0.354
4	0.46	1.10	1.88	0.076	0.28	1.90	0.65	0.522
5	0.90	1.45	2.79	0.012^*^	0.57	2.59	0.99	0.336
6	0.85	0.92	4.12	0.001^**^	0.52	1.62	1.44	0.166
7	0.71	0.95	3.37	0.003^**^	0.75	1.59	2.12	0.047^*^
8	1.14	1.58	3.21	0.005^**^	0.94	2.44	1.72	0.101
9	1.52	1.32	5.16	0.000^***^	1.37	2.51	2.43	0.025^*^
10	0.85	1.40	2.70	0.014^*^	0.78	2.48	1.42	0.173
11	0.99	1.45	3.04	0.007^**^	1.06	2.98	1.59	0.129
12	1.12	0.95	5.28	0.000^***^	0.93	1.28	3.26	0.004^**^
13	0.80	1.12	3.19	0.005^**^	0.82	1.76	2.09	0.051
14	1.26	1.88	3.00	0.007^**^	1.39	2.99	2.09	0.051
15	0.56	1.56	1.60	0.126	1.12	2.67	1.88	0.076
16	0.57	1.03	2.48	0.023^*^	0.78	1.98	1.77	0.093

* P < 0.05;

** P < 0.01;

*** P < 0.001.

There were no significant differences found in any of the channels between the mean oxyHb changes during neutral and negative picture presentation.

#### oxyHb changes during the n-back task

The results of the individual analysis of the n-back task were as follows. In the negative 1-back, 11 of 20 participants (55%) exhibited a significant oxyHb increase while one participant (5%) showed a significant oxyHb decrease. In the negative 3-back, a significant oxyHb increase was also observed in 11 of 20 participants (55%) while significant oxyHb decreases occurred in two participants (10%). Table 4 displays the results of the negative 1-back and negative 3-back tasks. In contrast, there were no participants who showed significant oxyHb increases or decreases in either neutral n-back task.

**Table 4:** **Channels that showed significant oxyHb changes during the n-back task (individual analysis)**.

**Participant no.**	**Negative 1-back**		**Negative 3-back**	
	**Activation**	**Deactivation**	**Activation**	**Deactivation**
1	9	-	-	-
2	-	10/11	-	11
3	-	-	-	-
4	11/13/14	-	-	-
5	-	-	-	4/6/7/13/14/16
6	-	-	1–3/5/7/9/11/13	-
7	-	-	1–7/9–16	-
8	1/9	-	1/2	-
9	-	-	-	-
10	-	-	-	-
11	-	-	7/11/13	-
12	2/5–12/14–16	-	-	-
13	3/5/8/10/12–15	-	13	-
14	4/6/7/9/12/13	-	5–7/9–12	-
15	2–6/8/10/11/13/14/16	-	1–4/7/9	-
16	1/4/5	-	3/4/6/10/11/16	-
17	11/13–16	-	-	-
18	-	-	1/3/4/6/12–16	-
19	1/2/4/7/8/11–15	-	16	-
20	3/6/7/9/10/12/13/16	-	1/12/16	-

Numerical numbers indicate channel numbers. The mark “-” indicates no significant oxyHb changes observed in any of the channels. The results of the neutral 1-back and neutral 3-back were omitted because there were no significant oxyHb changes observed.

**Figure 3:**
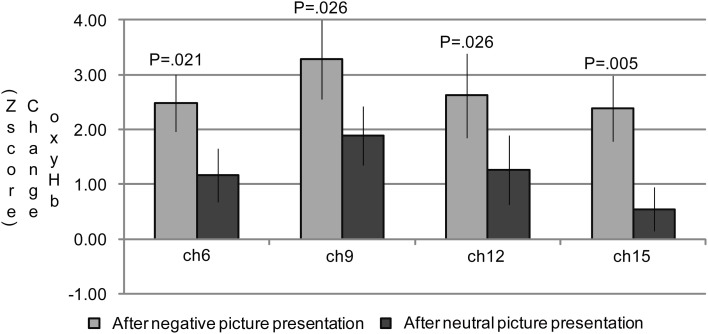
**The oxygenated hemoglobin (oxyHb) changes in the channels where the significant main effects of emotional valence were observed during the n-back task period.** In channels 6, 9, 12, and 15, significantly more oxyHb increases were observed during the n-back period after a negative picture presentation compared to that after a neutral picture presentation.

In the group analysis of the n-back task, a 2 (emotional valence: neutral or negative) × 2 (cognitive load: 1-back or 3-back) repeated measures ANOVA indicated significant main effects of emotional valence in channels 6 [*F*_(1, 19)_ = 6.31; *P* = 0.021], 9 [*F*_(1, 19)_ = 5.84; *P* = 0.026], 12 [*F*_(1, 19)_ = 6.35; *P* = 0.021], and 15 [*F*_(1, 19)_ = 12.05; *P* = 0.003] (Table 5; Figure 3). A Bonferroni *post-hoc* analysis revealed significantly more activation in the n-back task after negative picture presentations than in the n-back task after neutral picture presentation in all of the channels. No effects of cognitive load were found in any of these channels. In addition, no interaction effect of emotional valence or cognitive load was found.

**Table 5:** **The results of 2 (emotional valence) × 2 (cognitive load) repeated measures ANOVAs of the oxyHb changes during the n-back task (group analysis)**.

**Channel #**	**Emotional valence**		**Cognitive load**		**Interaction**	
	***F*_(1, 19)_**	***P***	***F*_(1, 19)_**	***P***	***F*_(1, 19)_**	***P***
1	0.79	0.386	0.34	0.569	1.10	0.307
2	1.27	0.275	0.03	0.861	0.02	0.889
3	2.04	0.170	0.10	0.752	0.04	0.841
4	0.59	0.450	0.01	0.909	0.73	0.405
5	0.48	0.496	0.08	0.787	1.30	0.268
6	6.31	0.021^*^	1.16	0.300	0.41	0.531
7	3.93	0.062	0.06	0.805	0.10	0.753
8	0.76	0.393	0.60	0.449	0.01	0.909
9	5.84	0.026^*^	0.00	0.949	2.50	0.130
10	1.36	0.258	1.73	0.205	0.14	0.709
11	2.32	0.144	1.04	0.322	0.18	0.674
12	6.35	0.021^*^	0.03	0.866	0.00	0.976
13	2.32	0.144	0.00	0.960	0.02	0.899
14	2.83	0.109	0.03	0.861	0.02	0.905
15	12.05	0.003^**^	0.00	0.957	0.00	0.988
16	1.54	0.230	0.12	0.737	1.55	0.229

* P < 0.05;

** P < 0.01.

**Figure 4:**
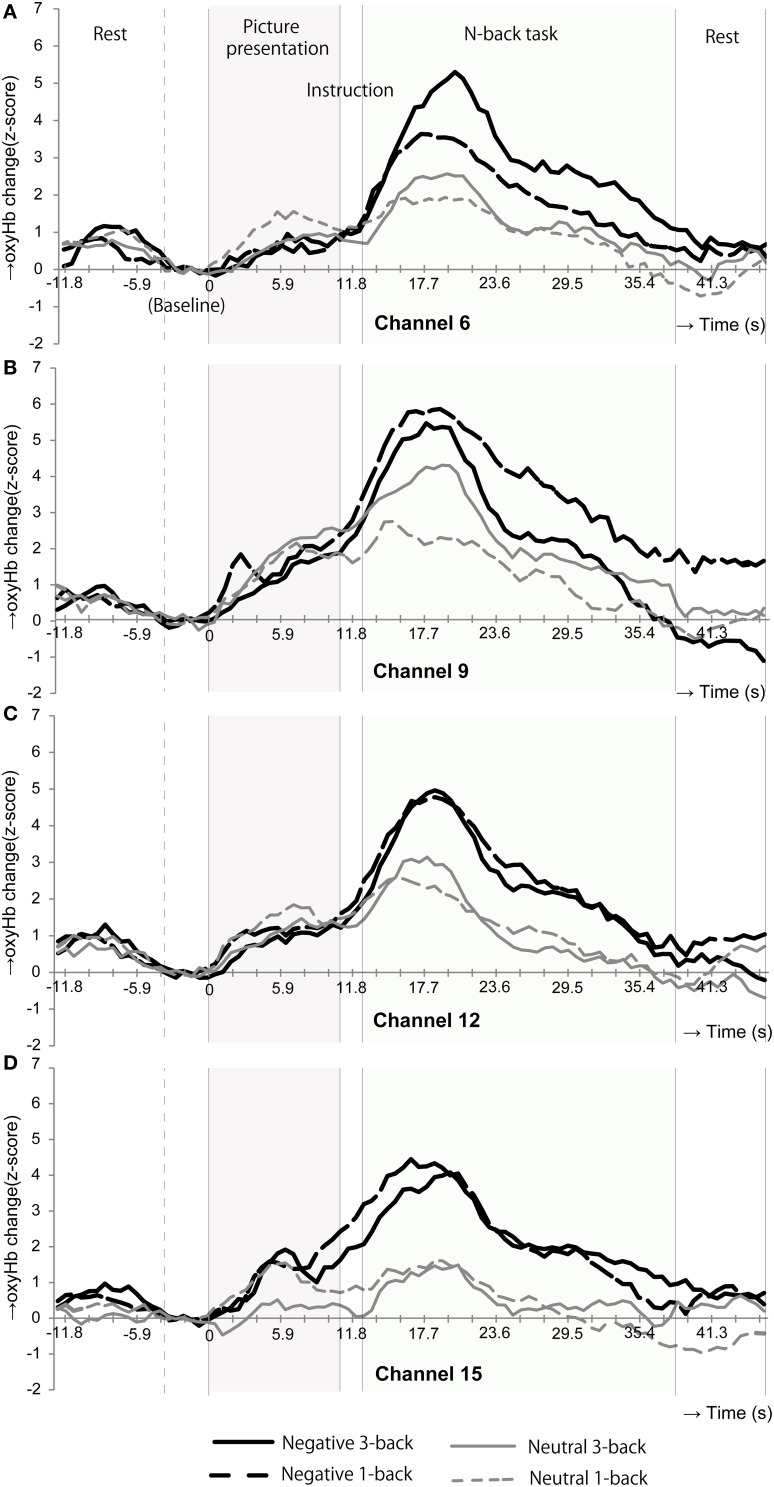
**Time courses of the oxyHb changes in the channels where the oxyHb changes in emotional valences were significantly different during the n-back task period.** In channels 6**(A)**, 9**(B)**, 12**(C)**, and 15**(D)**, the oxyHb changes were dramatically increased at the beginning of the n-back period after negative picture presentation.

Figure 4 shows the time courses of oxyHb changes in channels 6, 9, 12, and 15 where the significant main effects of emotional valence were observed during the n-back task period. Each waveform represents a time course of one trial. It was constructed by calculating a grand average of oxyHb changes in the neutral 1-back, neutral 3-back, negative 1-back, and negative 3-back. A large difference in activation was observed between emotional valences around 6 s after the n-back task in all channels.

## Discussion

The present NIRS study examined the neural correlates of the cognitive control of emotion in prefrontal regions. In the experiment, task-irrelevant emotional stimuli were presented prior to a working memory task in order to produce an emotional distraction. Participants observed a series of two negative or two neutral pictures and then conducted a 1-back or 3-back task. The oxyHb changes during the picture presentation period and during the n-back period were individually analyzed in order to separate cognitive and emotional processing.

During picture presentation, there was no significant difference in brain activations between the neutral and negative stimulations though more channels showed significant activation in the neutral compared to the negative stimulation compared to baseline in group analysis (Table 3). This was probably because the variances of the oxyHb change of channels were relatively larger in negative compared to neutral stimulation though there was no large difference in the mean oxyHb values between the emotional valences (mean ± *SD*; neutral 0.82 ± 1.29, negative 0.83 ± 2.21). A large individual difference of prefrontal activation in response to negative (unpleasant) pictures is also observed in the findings of Hoshi et al. (2011). In their NIRS study, participants were exposed to a negative or positive (pleasant) IAPS picture for 6 s following a resting period of 14 s in order to examine the prefrontal processing of emotion. In response to negative pictures, seven of 14 participants (50%) indicated an oxyHb increase and six participants (42.9%) showed an oxyHb decrease. In a similar way, our findings showed that 45% of participants showed an oxyHb increase and 30% of the participants showed an oxyHb decrease in response to negative pictures (Table 2). In Hoshi et al. (2011), they then conducted group analysis by using only the data that were rated as “1” (the most unpleasant) for the pictures in order to extract responses of strong negative emotion. Consequently, the group analysis revealed significant activation in the bilateral VLPFC. By adopting only the data rated as “1,” individual differences in the oxyHb changes for the negative picture probably became smaller as the mean oxyHb values became larger. Doing this would mean that a statistically significant activation might be observed for negative pictures. In this way, by reducing individual differences in the responses for emotional pictures, prefrontal processing of emotion can be captured by NIRS. A large individual difference is presumably caused by differences of personality traits and brain structures and so on. Especially, in response to negative pictures, various personality traits seem to be related to a brain activity such as amygdala reactivity to negative picture presentation and amygdala-prefrontal connectivity. For example, it is reported that the right amygdala-DMPFC connectivity for angry and fearful compared to neutral faces was positively correlated with neuroticism scores (Cremers et al., 2010). Further research regarding individual differences in emotional processing caused by variations in personality traits is necessary.

Though no significant differences in oxyHb changes between neutral and negative stimulations were exhibited during the picture presentation, significant differences by emotional valence were observed during n-back task in channel 6, 9, 12, and 15 (Table 5). These channels were placed close to Fp1 (channel 12), Fpz (channel 9), Fp2 (channel 6), and F7 (channel 15) in the International 10/20 System. According to Okamoto et al. (2004), Fp1, Fpz, and Fp2 are located in the left and right superior frontal gyrus and F7 is in the left inferior frontal gyrus. Among these regions, the activation in the left inferior frontal gyrus is frequently observed as the result of the cognitive control of emotion (Cromheeke and Mueller, 2013). Thus, activation in channel 15 was presumably led by emotion regulation. Greater activation in the left hemisphere, particularly the left inferior frontal gyrus, was observed in our study. However, we did not observe the right DLPFC activation which is also relatively frequently activated by emotion regulation (Lévesque et al., 2003; Dieler et al., 2010; Cromheeke and Mueller, 2013). For example, in Van Dillen et al. (2009), the right DLPFC activation was found after the onset of the complex arithmetic task following negative picture presentations. The reason why we observed greater activation in the left hemisphere is unclear. However, it may be partially because we employed a verbal n-back task, processing of which is known to be associated mainly with the left hemisphere (Owen et al., 2005). The difference in the cognitive task may be one factor in determining which regions are susceptible to emotional effects.

The NIRS system is unable to measure the entire region of MPFC. However, channel 12 (Fp1), 9 (Fpz), and 6 (Fp2) possibly measure the oxyHb change in the anterior part of the MPFC. MPFC (Phan et al., 2002) are the regions commonly activated in emotional responses. Thereby, the additional activations during the n-back task after negative stimulation in these channels might simply represent emotional responses induced by picture presentation, considering the delay of hemodynamic responses. On the one hand, the MPFC is also known to be associated with emotional regulation (Davidson et al., 2000). Thereby, it is possible that the NIRS system captured the cognitive control processing occurred in the anterior part of MPFC in our study. Moreover, looking at the time courses of the oxyHb changes (Figure 4), during the picture presentation period, both the neutral and negative picture presentation increased the oxyHb change in these channels. Only when participants performed the n-back task, prefrontal responses in these channels began to differentiate: even more rapid oxyHb increase was occurred in negative 3-back and negative 1-back compared to neutral 3-back and neutral 1-back. This rapid oxyHb increase in negative 3-back and negative 1-back was reached approximately at 6 s after the n-back task onset. This rapid activation seems to represent the effect of the n-back task implementation and not a simple emotional response. This activation appears to include both emotional and cognitive processing and implies the possibility of the cognitive control processing of emotion.

We observed the effect of negative stimulation only in brain activations during the cognitive task while there was no emotional effect observed in behavioral performance. This is because brain activation might be caused by a compensatory effort of the brain according to the processing-efficiency hypothesis (Eysenck et al., 2007). The hypothesis suggests that highly-anxious people require greater cerebral activation, the compensatory effort, for cognitive control in order to maintain the same level of performance as non-anxious people. The impairment of behavioral performance is compensated for if resources in working memory are available (Fales et al., 2008; Basten et al., 2012). Thus, there seems to be a potential ability of a healthy brain to maintain behavioral performance at a high level under an anxious state. In our study, negative pictures yielded an unpleasant emotional state, which is presumably similar to anxiety. Even under such an unpleasant state, the behavioral performance was maintained probably because we employed participants who had sufficient working memory resources. However, it is important to examine if similar tendencies can be observed using other cognitive tasks.

Our findings revealed a significant effect of cognitive load on reaction times, with responses being significantly faster during the 1-back task than during the 3-back task (mean reaction times: 1-back, 246.32 ms; 3-back, 363.77 ms, *P* < 0.001). Thus, the two cognitive loads actually differed in difficulty level. However, cognitive load did not significantly affect accuracy on the n-back task, and although changes in oxyHb were greater in negative and neutral 3-back tasks than in negative and neutral 1-back tasks in some channels (Figure 4), group analysis did not reveal any effect of cognitive load on changes in oxyHb during the n-back task (Table 5). In contrast, Van Dillen et al. (2009) reported that accuracy was better and reaction times faster for a simple arithmetic task than for a complex one. At the same time, brain activity was greater for the complex arithmetic task than for the simple one after negative stimulation. Significant effects of cognitive load on reaction times and brain activity may therefore be observed by employing tasks with cognitive loads that differ more greatly than ours did.

In sum, the present NIRS study clearly indicated additional activation in channels 6, 9, 12, and 15 during the n-back after negative stimulation. It thus indicated the ability of the NIRS system to capture emotion-related processing, though further research is necessary for the interpretation of this activation and the roles of personal traits for individual differences of brain activity, particularly adopting other experimental designs, cognitive tasks, and self-report questionnaires, for example. It is interesting that the emotional effect was observed only in brain activations during the n-back task but not in behavioral performance because it implies that brain activation could be an effective index of emotion regulation. By adopting the NIRS system, the study of emotion regulation can be more easily studied in various participants, such as children and psychiatric participants.

### Conflict of interest statement

The authors declare that the research was conducted in the absence of any commercial or financial relationships that could be construed as a potential conflict of interest.
